# Tick-borne Pathogens in Northwestern California, USA

**DOI:** 10.3201/eid2003.130668

**Published:** 2014-03

**Authors:** Daniel J. Salkeld, Stephanie Cinkovich, Nathan C. Nieto

**Affiliations:** Stanford University, Stanford, California, USA (D.J. Salkeld);; Northern Arizona University, Flagstaff, Arizona, USA (S. Cinkovich, N.C. Nieto)

**Keywords:** Borrelia burgdorferi, Borrelia miyamotoi, California, tick-borne disease, Ixodes pacificus, western black-legged tick, Lyme disease, vector-borne infections, spirochete, bacteria

**To the Editor:** In northwestern California, USA, the western black-legged tick, *Ixodes pacificus*, is a known vector of *Borrelia burgdorferi*, the spirochete that causes Lyme disease. *B. miyamotoi,* which is more closely related to spirochetes that cause relapsing fever, has also been detected in 2 locations in California (*1*,*2*) and has recently been implicated as a human pathogen in the northeastern United States (*3*,*4*). Other studies may have unintentionally included *B. miyamotoi* infections among measures of *B. burgdorferi* if the diagnostics were for spirochetes (e.g., direct fluorescent antibody tests or dark-field microscopy) or genetically targeted for *Borrelia* spp. (*5*).

To investigate *Borrelia* spp. ecology in California, we collected adult *I. pacificus* ticks by dragging a 1-m^2^ white flannel blanket along vegetation and/or leaf litter in 12 recreational areas in the San Francisco Bay area during January–May 2012 (Table). Habitat varied from chaparral and grassland to coastal live oak woodland. Ticks were pooled for examination by quantitative PCR (qPCR) for the presence of *Borrelia* spp. We interpreted the prevalence of *Borrelia* spp. from positive pools as the minimum infection prevalence (i.e., assuming 1 positive tick/positive pool). DNA was extracted from ticks by using the DNeasy Blood and Tissue Kit (QIAGEN, Valencia, CA, USA) according to the manufacturer’s protocols and then stored at −20°C until use. DNA was analyzed by qPCR, with use of primer and fluorescent hybridization probes previously developed to differentiate *Borrelia* spp. spirochetes (*5*). To identify the *Borrelia* spp. genotype, we attempted to sequence the 16S–23S (*rrs-rrlA*) intergenic spacer of each sample positive by qPCR (*8*). The nested PCR product was further purified by using the QIAquick Kit (QIAGEN) and then sequenced (Environmental Genetics and Genomics Laboratory, Northern Arizona University, Flagstaff, AZ, USA; www.enggen.nau.edu/dna.html) by using capillary Sanger sequencing on an ABI 3730 sequencer (Life Technologies, Grand Island, NY, USA). BLAST (http://blast.ncbi.nlm.nih.gov/Blast.cgi) was used to compare each sequence to other *Borrelia* spp. sequences available from GenBank.

**Table:** *Borrelia* spp. infection prevalence among adult *Ixodes pacificus* ticks in northwestern California, USA, January–May 2012*

Location, County (reference)	No. *Borrelia* spp. ticks infected/total (%)				
	*B. burgdorferi* sensu stricto	*B. burgdorferi* sensu lato	*B. miyamotoi*	Unsequenced species	All species
Jasper Ridge Biologic Preserve, San Mateo				1/32 (3.1)	1/32 (3.1)
Pulgas Ridge OSP, San Mateo				2/118 (1.7)	2/118 (1.7)
Thornewood OSP, San Mateo†	1/156 (0.6)	2/156 (1.3)	2/156 (1.3)	4/156 (2.6)	9/156 (5.8)
Thornewood OSP, San Mateo‡					0/9 (0)
Windy Hill OSP, San Mateo†	2/120 (1.7)			1/120 (0.8)	3/120 (2.5)
Windy Hill OSP, San Mateo§	2/122 (1.6)	3/122 (2.5)	1/122 (0.8)	2/122 (1.6)	8/122 (6.6)
Wunderlich County Park, San Mateo					0/15 (0)
Foothills Park, Santa Clara					0/13 (0)
Henry W. Coe State Park, Santa Clara			3/132 (2.3)		3/132 (2.3)
Monte Bello OSP, Santa Clara	1/46 (2.2)			1/46 (2.2)	2/46 (4.3)
Sanborn County Park, Santa Clara			4/53 (7.5)		4/53 (7.5)
Sierra Azul OSP, Santa Clara			2/112 (1.8)		2/112 (1.8)
Los Trancos OSP, San Mateo and Santa Clara			1/58 (1.7)	1/58 (1.7)	2/58 (3.4)
Castle Rock State Park, Santa Cruz		1/51 (2.0)		2/51 (3.9)	3/51 (5.8)
Castle Rock State Park, Santa Cruz (*6*)					13/264 (4.9)
Tilden Regional Park, Contra Costa (*2*)	1/814 (0.1)		4/814 (0.5)		5/814 (0.6)
China Camp State Park, Marin		1/143 (0.7)	1/143 (0.7)	2/143 (1.4)	4/143 (2.8)
Hopland Research and Extension Center, Mendocino (*1**,**7**)*	4/282 (1.4)		2/282 (0.7)		
Total (this study)	6/1,108 (0.5)	7/1,108 (0.6)	14/1,108 (1.3)	16/1,108 (1.4)	43/1,108 (3.6)

*Data are from this study and from previously published research (indicated by reference no.). OSP, open space preserve. †Woodland. ‡Redwood. §Chaparral/grassland.

From a total of 1,180 adult ticks, we found 43 samples positive for *Borrelia* spp., resulting in a minimum infection prevalence of 3.6% (Table). We obtained intergenic spacer sequence data for 27 of the positive samples; 6 samples were *B. burgdorferi* sensu stricto, 7 were *B. burgdorferi* sensu lato (both on the basis of alignments of 816 bp), and 14 were *B. miyamotoi* (on the basis of alignments of 503 bp)*.* The *B. miyamotoi* sequences for our samples from California and those for isolates from the eastern United States (*9*) and Japan (*8*) formed a monophyletic clade that was oriented as a sister clade to the 3 *Borrelia* spp. that cause tick-borne relapsing fever in the United States (*B. hermsii, B. turicatae*, and *B. parkeri*).

We found borreliae-infected adult *I. pacificus* ticks at all 12 sites from which tick sample sizes exceeded 30. When the presence of *B. burgdorferi* sensu stricto or *B. burgdorferi* sensu lato was detected (4/12 sites each), prevalence was 0.6%–2.2% and 0.7%–2.5%, respectively. *B. miyamotoi* was detected at 7/12 sites, and prevalence ranged from 0.7% to 7.5%. A previous survey of *B. burgdorferi* in nearby Santa Cruz County recreational areas reported an infection prevalence of ≈6% among adult *I. pacificus* ticks (*6*); the study did not, however, differentiate between *Borrelia* spp. and therefore may have included *B. miyamotoi* among its prevalence measures (*5*). In our study, *B. burgdorferi* was found more frequently in woodland habitats, but it was also detected in a grassland–chaparral habitat several hundred meters from the nearest woodland. We did not detect *B. bissettii*, a species recently implicated as a human pathogen in Mendocino County, California (*10*). The high level of habitat variation in northwestern California presents a varied risk for *Borrelia*-associated tick-borne disease in humans because of diverse variations in vertebrate reservoir ecology, tick abundance, and human exposure to ticks. This variation emphasizes the need to understand the local epidemiology and ecology of a disease.

In adult *I. pacificus* ticks in the San Francisco Bay area, *B. miyamotoi* is as abundant as its congener *B. burgdorferi.* Human disease caused by *B. miyamotoi* infection has not been reported in California, and transmission efficiency of *B. miyamotoi* by *I. pacificus* ticks is unknown. However, it is possible that *B*. *miyamotoi* infections in ticks and humans have not been accurately diagnosed. We advocate for increased scrutiny of the eco-epidemiology of *B. miyamotoi* in human, tick, and possible vertebrate host populations in northwestern California.
